# Xyloglucan for the Treatment of Acute Gastroenteritis in Children: Results of a Randomized, Controlled, Clinical Trial

**DOI:** 10.1155/2016/6874207

**Published:** 2016-05-03

**Authors:** Cătălin Pleșea Condratovici, Vladimir Bacarea, Núria Piqué

**Affiliations:** ^1^Faculty of Medicine, “Lower Danube” University of Medicine & Pharmacy, Galati, Romania; ^2^University of Medicine & Pharmacy of Targu-Mures, Targu-Mures, Romania; ^3^Microbiology Department, Pharmacy Faculty, Universitat de Barcelona, Edificio A, Avenida Joan XXIII, 08028 Barcelona, Spain

## Abstract

*Background*. Xyloglucan, a film-forming agent, improves intestinal mucosa resistance to pathologic damage. The efficacy, safety, and time of onset of the antidiarrheal effect of xyloglucan were assessed in children with acute gastroenteritis receiving oral rehydration solution (ORS).* Methods*. This randomized, controlled, open-label, parallel-group, multicenter, clinical trial included children (3 months–12 years) with acute gastroenteritis of infectious origin. Children were randomized to xyloglucan and ORS, or ORS only, for 5 days. Diarrheal symptoms, including stool number/characteristics, and safety were assessed at baseline and after 2 and 5 days and by fulfillment of a parent diary card.* Results*. Thirty-six patients (58.33% girls) were included (*n* = 18/group). Patients receiving xyloglucan and ORS had better symptom evolution than ORS-only recipients, with a faster onset of action. At 6 hours, xyloglucan produced a significantly greater decrease in the number of type 7 stools (0.11 versus 0.44; *P* = 0.027). At days 3 and 5, xyloglucan also produced a significantly greater reduction in types 6 and 7 stools compared with ORS alone. Xyloglucan plus ORS was safe and well tolerated.* Conclusions*. Xyloglucan is an efficacious and safe option for the treatment of acute gastroenteritis in children, with a rapid onset of action in reducing diarrheal symptoms. This study is registered with ISRCTN number 65893282.

## 1. Introduction

Acute gastroenteritis, characterized by the onset of diarrhea with or without vomiting, is an extremely common problem in childhood and the second leading cause of morbidity and mortality worldwide, particularly in the first 3 years of life [1, 2], with the majority of deaths concentrated in 35 “low income” countries [3]. In Europe, it is usually, although not always, a mild disease, and death is an exceptional outcome. However, gastroenteritis is associated with a substantial number of hospitalizations and high costs [1].

According to guidelines for the management of European children, rehydration is the key treatment and should be applied as soon as possible [1]. Reduced osmolality oral rehydration solution (ORS) should be used, and it should be offered* ad libitum* and rapidly (i.e., within 3-4 hours). Regular feeding should not be interrupted and should be maintained following initial rehydration. Drugs are generally not necessary; however, selected probiotics may reduce the duration and intensity of symptoms. Antibiotic therapy is not needed in most cases of acute gastroenteritis and may induce a carrier status in cases of* Salmonella* infection. Antibiotic treatment is effective mainly in shigellosis and in the early stage of* Campylobacter* infection. According to these guidelines, other drugs may be effective but require further investigation [1]. In this scenario, there is scope to develop new products that could be used in combination with ORS to reduce the duration and number of symptoms, with a good safety profile [4, 5]. Food supplements and medical devices can provide these requirements, although randomized, controlled studies are still needed [5].

A new class of products, defined as “mucosal protectors” has been developed for use in gastroenteric diseases. These products form a bioprotective film on the intestinal mucosa, improving the resistance of the mucosa to pathologic aggression and helping to restore normal function [6, 7]. Among these film-forming products, gelatin tannate, gelatin, and xyloglucan are currently being studied and used for gastroenteric disorders, although further randomized studies are needed to completely assess the efficacy of these products in acute diarrhea or acute gastroenteritis in different types of patients [6–8].

In this regard, a medical device containing xyloglucan, extracted from the seeds of the tamarind tree (*Tamarindus indica*), has been developed and has recently received European approval (MED class III) for restoring the physiological functions of the intestinal walls. Formulated as capsules for adults and powder for pediatric use, the xyloglucan-containing product has been developed specifically for the control and reduction of symptoms related to diarrheal events of different etiologies, such as abdominal tension and frequent emissions of feces. Xyloglucan, ingested in appropriate amounts, forms a protective biofilm on the intestinal mucosa that improves the resistance of the mucosa to pathologic aggression and helps to restore its normal function. In particular, xyloglucan has been shown to increase the Transepithelial Electrical Resistance (TEER), an index of the function of the mucosal tight junctions, in Caco 2 monolayers, thus confirming its ability to counteract mucosal permeability and leakage which is typical of diarrhea. In the same* in vitro* model, xyloglucan was able to restore normal TEER values after leakage induced with exposure to* E. coli* [9]. The same properties of xyloglucan have also been demonstrated* in vivo* by restoring the huge mucosal leakage induced by intraperitoneal injection of* E. coli* lipopolysaccharide (LPS, 1 mg/kg) in adult rats [9].

In a previous randomized, multicenter, open-label study, we have demonstrated the efficacy and safety of xyloglucan in adult patients with acute diarrhea. In comparison with two widely used antidiarrheal products,* Saccharomyces boulardii*, containing the yeast probiotic* S. boulardii*, and diosmectite, an absorbent activated natural aluminosilicate clay, xyloglucan exhibited a faster onset of action in terms of a reduction in the mean number of types 6 and 7 stools (the most dehydrating type of stools), particularly during the first hours posttreatment. Xyloglucan was also the most efficient treatment in reducing the percentage of patients with nausea and abdominal pain throughout the study period, with an excellent safety profile [10].

Based on these favorable results in adults, and considering the need to develop new products for the management of acute gastroenteritis in children [1, 4, 5], we designed the present randomized, controlled, open-label, parallel-group, multicenter, clinical trial to assess the efficacy and safety of xyloglucan in pediatric patients with acute gastroenteritis.

## 2. Patients and Methods

### 2.1. Study Design

This randomized, controlled, open-label, parallel-group, multicenter, clinical trial was performed to evaluate the efficacy and safety of xyloglucan plus ORS, in comparison with ORS alone in pediatric patients (aged from 3 months to 12 years) with acute gastroenteritis.

The study protocol was approved by the Ethical Committee for Scientific Research of the “Targu-Mures” University of Medicine and Pharmacy, Romania (number 60, dated 8 July 2012) and procedures were carried out in accordance with the ethical standards of the Declaration of Helsinki (revised 2000). Written informed consent was obtained for all children: in all cases, from the child's parents or legal guardians and directly also from children aged 7–12 years. Patients were recruited in different Romanian outpatient general practitioner medical offices during routine clinical practice.

### 2.2. Inclusion/Exclusion Criteria

Children aged between 3 months and 12 years, diagnosed with acute gastroenteritis (acute diarrhea) of infectious origin with absent or mild-moderate dehydration that could be treated with ORS and diet (without requiring antibiotic therapy) in an outpatient setting, were included in the study. Acute diarrhea was defined as the occurrence of ≥3 stools per day graded as 6 or 7 on the Bristol Stool Scale (BSS) [11, 12] during a period shorter than 72 hours. The diagnosis was made according to the investigators' judgment based on the clinical picture including objective (stools, vomiting, and fever) and subjective (nausea, abdominal pain, and bloating) symptoms.

Potential participants were excluded in cases of infantile colic, diarrhea due to milk/protein intolerance, severe dehydration requiring intravenous rehydration, need for hospitalization, use of antidiarrheal treatment (before baseline or during the study period), and chronic or toxic diarrhea and in cases where it was impossible to follow up the patient for more than 12 hours.

### 2.3. Treatment and Randomization

Patients were randomly assigned to receive xyloglucan and ORS or ORS alone at a ratio of 1 : 1. Xyloglucan was administered in the form of oral sachets (containing xyloglucan, gelatin of porcine origin, corn starch, and magnesium stearate). ORS (Humana Elektrolyt Banane, Humana GmbH, Germany) was administered as a powder for oral solution (containing sodium- and potassium-chloride and glucose).

The allocation of subjects to a treatment group was determined by a computer-generated randomization list, using a pseudorandom number generator. The list contained 36 randomization positions, from 1 to 36, with an active/control ratio of 1 : 1. Each study site received a sublist of randomization positions.

### 2.4. Study Procedure

During the first enrolment visit (visit 0), patients were randomized into two groups (xyloglucan or ORS) to receive a 5-day treatment (one sachet every 8 hours for children younger than 3 years and 2 sachets every 8 hours in the case of xyloglucan in those older than 3 years, while ORS was prescribed according to leaflet provisions and medical judgment), with the first dose being administered at the time of recruitment (visit 0). In order to assess adherence to treatment, parents or legal guardians recorded the use of study medication on the diary card and were instructed to return all packages of the used and unused product at visit 1 (performed 2 days after baseline) and at visit 2 (5 days after baseline).

During the baseline visit, demographic, anthropometric (weight, height, and body mass index), and clinical data (including vital signs, comorbidities, and symptomatology of acute gastroenteritis during the previous 3 days and at the recruitment visit) were recorded. Clinical symptoms of acute gastroenteritis were assessed by patient interviews and exploration and included nausea, vomiting, anorexia, abdominal pain, flatulence, stools (type, number, and duration of diarrhea, presence of blood/mucus/pus in feces), fever, dehydration (abnormal skin turgor, weight decrease), and signs of peritonitis and/or sepsis. Stool consistency was classified using the 7-point BSS [11, 12].

During the baseline visit (visit 0), parents or legal guardians also received an* ad hoc* questionnaire, included in the patient's daily diary, to assess the consistency of stools and diarrheal symptoms (according to the BSS) at 1, 3, 6, 12, and 24 hours following administration of the first dose. Stool emissions (including the number of emissions/day), with mucus and/or blood, were recorded and the consistency of each stool was assessed using the BSS (type 1 corresponds to separate hard lumps, like nuts, while type 7 corresponds to watery, no solid pieces, entirely liquid) [11, 12]. The presence of subjective symptoms such as nausea, vomiting (including number of vomits/day), abdominal pain, and flatulence was also recorded in the* ad hoc* questionnaire.

At visit 1 (2 days after the baseline visit) and visit 2 (5 days after baseline) the investigators reviewed the patient's daily diary. During these visits, symptoms and clinical signs were recorded and symptom assessment was also performed by patient interviews and exploration.

The occurrence of adverse events was assessed at all study visits and by follow-up phone calls (performed 10 days after study completion). All these data were transferred into the electronic patient's case report form.

### 2.5. Outcome Measures

The primary efficacy variable was the variation in the number of type 7 stools and types 6 and 7 stools during the 5-day treatment and the rapidity of action of the studied products in the intention-to-treat (ITT) population (all randomized patients who had at least one posttreatment measurement). The primary safety variable was the prevalence of adverse events in both groups of patients in the safety population (all patients who received at least one dose of the investigational product).

### 2.6. Statistical Analyses

Sample size calculation was based on the primary variable of the study, that is, to demonstrate a difference in the number of dehydration stools between both groups, based on the number of stools obtained in previous similar studies [13]. Based on this, the objective was to observe an approximate reduction of 3.8 in the absolute number of stools.

The sample size (*n* = 36, *n* = 18 in each group) was calculated to have a 90% power to demonstrate, with 95% probability, differences (a noninferiority margin difference between the group proportions of 0.1100) in the evolution of the number of types 6 and 7 stools during the study period. Sample size calculation was also based on results obtained in previous similar studies [13].

Descriptive analyses (within-patient *n*, mean, median, standard deviation, minimum, and maximum) were performed for quantitative variables and frequency counts by category were calculated for qualitative variables. Following the results of normality tests (Kolmogorov-Smirnov test), data obtained at the three visits (baseline, 2 and 5 days) were compared by means of Friedman's ANOVA and Kendall's coefficient of concordance for nonparametric dependent data. Comparisons of data obtained at a specific visit between the two treatments were performed by means of Mann-Whitney *U* test for nonparametric and independent data. Two-sided *P* values were obtained and statistically significant results were declared if *P* < 0.05. Statistical analyses were performed using IBM SPSS 19 software for Windows.

## 3. Results

### 3.1. Patient Characteristics

A total of 36 patients were included in the study (18 in each group). All randomized patients had at least one posttreatment measurement and received at least one dose of the product; thus the ITT and safety populations coincided.

Table 1 shows the demographic and clinical characteristics of patients. Girls accounted for 58.3% of the total study population. Identical numbers of girls and boys were included in the active group (50.00%; *n* = 9), whereas more girls were included in the control group (66.67%; *n* = 12) (Table 1).

The overall mean age of children was 4.33 ± 3.80 years (4.72 ± 4.33 in the active group and 3.94 ± 3.26 in the control group) (Table 1). By age ranges, children aged 1–5 years were the most prevalent group (38.89% in the active group; 55.56% in the control group; 47.22% in the total sample), followed by those aged 5–10 years range (22.22% versus 27.78%, and 25.00% in the total sample) and >10 years (22.22% vs. 5.56%, and 13.88% in the total sample) and ≤1 year (16.67% versus 11.11%, and 13.88% in the total sample) (Table 1).

At baseline, the majority of patients had normal hydration status (61.11% versus 72.22%) and the remainder presented with mild dehydration, while the majority of patients in both groups had temperatures ranging from 37°C to 38°C (27.78% versus 33.33%) or higher than 38°C (22.22% versus 27.78%) (Table 1). No patient required hospitalization or intravenous fluids.

Vital signs, including heart rate and breath rate, were within the normal values in all patients, while no relevant comorbidities were present.

### 3.2. Reduction of Diarrheal Symptoms

**Figure 1:**
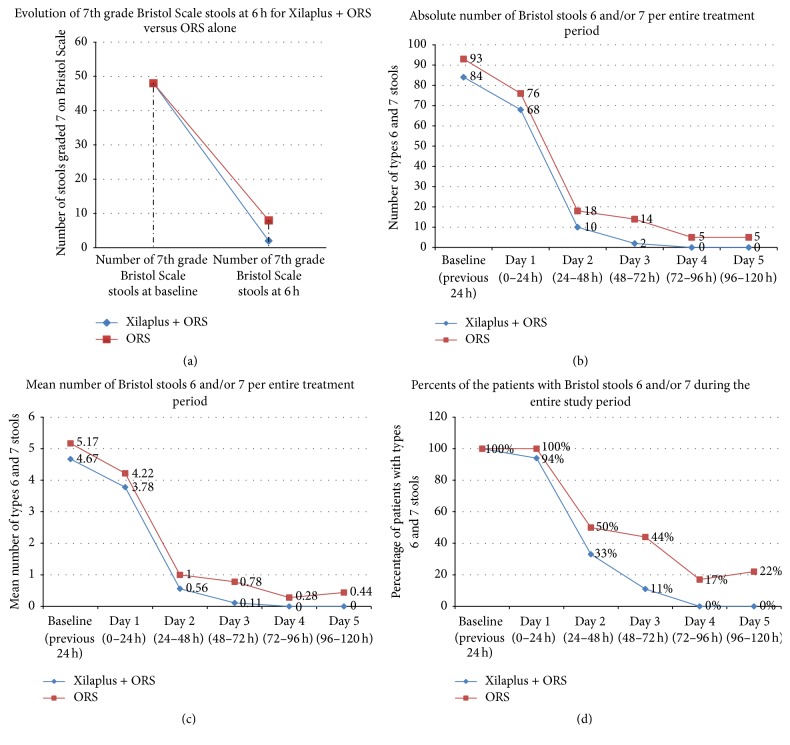
Evolution of types 6 and 7 Bristol Scale stools in both groups. (a) Evolution of the absolute number of type 7 stools during the first 6 hours. (b) Evolution of absolute number of types 6 and 7 stools during the study period. (c) Evolution of mean number of types 6 and 7 stools during the study period. (d) Evolution of the percentage of patients with types 6 and 7 stools during the study period.

During the first 6 hours of treatment, the group treated with xyloglucan and ORS showed a faster onset of action and improvement of diarrheal symptoms, measured as absolute number of type 7 stools, compared with the control group. Consequently, in the active group the highest reduction in the number of type 7 stools was observed at 6 hours (active group: 48 stools at baseline to 2 stools at 6 hours; control group: 48 stools at baseline to 8 stools at 6 hours), with an effect that was statistically significant compared with the control group (*P* = 0.027) (Figure 1(a)). The same significant difference was observed considering the evolution of the mean number of type 7 stools (active group: from 2.67 to 0.11; control group: from 2.67 to 0.44).

Considering types 6 and 7 stools, we observed a more pronounced reduction in the group treated with xyloglucan and ORS in comparison with the control group, in terms of absolute number of types 6 and 7 stools (Figure 1(b)) and mean number of types 6 and 7 stools (Figure 1(c)). In the active group, the number of types 6 and 7 stools decreased from 93 stools at baseline to 68 at day 1, 10 at day 2, and 2 at day 3, disappearing at days 4 and 5, while in the control group at days 4 and 5, types 6 and 7 stools were still present (Figure 1(b)). Statistically significant differences in the number of types 6 and 7 stools between active and control group were observed at day 3 (*P* = 0.026) and day 5 (*P* = 0.034). The same trend was observed in terms of the evolution of mean number of types 6 and 7 stools (Figure 1(c)) and also considering the percentage of patients with types 6 and 7 stools at each study visit (Figure 1(d)). In comparison with the control group, the percentage of patients with types 6 and 7 stools was always lower in the active group from day 1 to day 5, with the difference being statistically significant at days 3 (*P* = 0.026; Pearson's Chi-squared test) and 5 (*P* = 0.034; Pearson's Chi-squared test) (Figure 1(d)).

We also observed statistically significant differences in the duration of diarrhea between both groups, being 3 days for the active group and 5 days for the control group (*P* = 0.042).

### 3.3. Reduction of Nausea, Vomiting, and Flatulence

**Figure 2:**
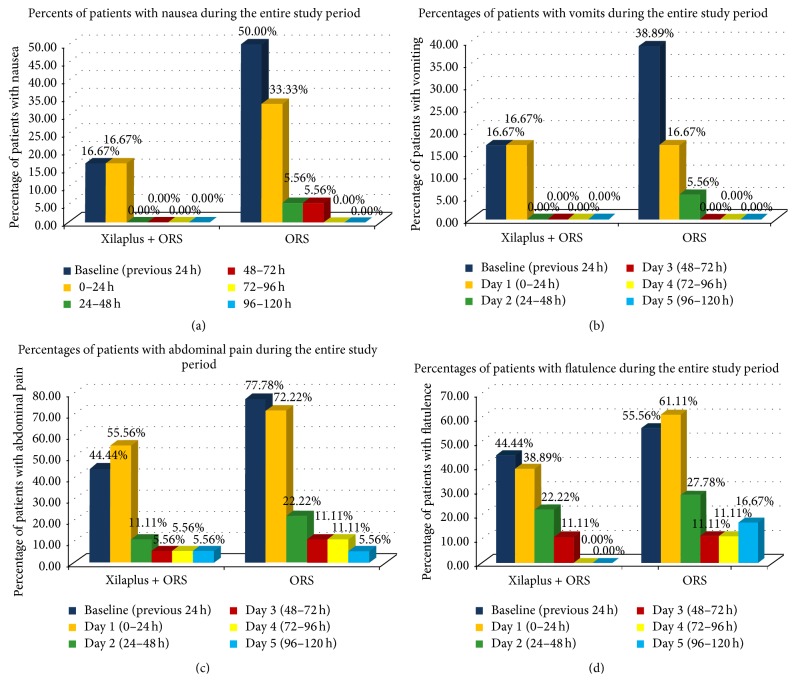
Evolution of clinical symptoms of gastroenteritis during the study period in both groups. (a) Evolution of the percentage of patients with nausea. (b) Evolution of the percentage of patients with vomiting. (c) Evolution of the percentage of patients with abdominal pain. (d) Evolution of the percentage of patients with flatulence.

Despite some between-group baseline differences, a trend to higher effect of xyloglucan and ORS in reducing the percentage of patients with nausea was observed throughout the study period, although no statistically significant differences were observed in comparison with the control group. We note that, in the active group, the percentage of patients without nausea was achieved after 24 hours while, in the control group, disappearance of nausea occurred at 72 hours after baseline (Figure 2(a)). Similarly, the disappearance of vomiting occurred earlier in the active group (after 24 hours) (Figure 2(b)). We also noted an earlier reduction in the percentage of children with abdominal pain (Figure 2(c)) and also with flatulence (Figure 2(d)) in the active group.

### 3.4. Safety of Treatment

Both treatments were safe and well tolerated, with no adverse events being reported during the study period.

## 4. Discussion

Rehydration is the key treatment for acute gastroenteritis in children and should be applied as soon as possible [1] to avoid risks and complications, such as life-threatening dehydration, electrolyte disturbances, disturbed digestion, and absorption of nutrients with nutritional deterioration, leading to the need for enteral/parenteral rehydration and consequent hospitalization [1, 2, 14]. For this reason, interventions which can increase the efficacy of ORS deserve special consideration.

In the present study, we have demonstrated that, compared with ORS alone, ORS administrated in combination with xyloglucan produced a significant reduction in BSS types 6 and 7 stools as early as 6 hours posttreatment. This faster onset of action supports the use of this combination to reduce the number of types 6 and 7 stools and associated complications in children.

These results are also in line with the findings obtained in a previous randomized clinical trial in adults with acute diarrhea [10]. In that study, during the first 24 hours of treatment, patients in the xyloglucan group showed a faster onset of action and improvement of diarrheal symptoms (measured as the absolute number of BSS types 6 and 7 stools), compared with patients receiving diosmectite or* S. boulardii*. Consequently, the highest reduction in the number of types 6 and 7 stools was observed at 6 hours in the xyloglucan group, an effect that was statistically significant compared with the diosmectite group (*P* = 0.031) [10].

It seems clear, therefore, that xyloglucan, in both children and adults, is able to stop dehydration by rapidly reducing the number of BSS types 6 and 7 stools. These results are also in line with findings from* in vitro* and* in vivo *studies, in which xyloglucan generated a protective pH-resistant biofilm in intestinal epithelial cells with antiabsorptive properties. By blending xyloglucan with natural gelatin type A, the biofilm improved cellular absorptive properties, forming a physical barrier that counteracted the effects of translocating microorganisms and toxins by reinforcing TEER. In that way, xyloglucan significantly reduced the damage to tight junctions and inflammatory immune response triggers [9].

Altogether, these results support the use of xyloglucan and other film-forming agents of the “mucoprotectors” family for the management of diseases that are accompanied by diarrhea, as in the case of gastroenteritis in children.

In an observational study in 239 children (aged from 3 months to 12 years) with acute diarrhea with two treatment arms (ORS alone or ORS plus another film-forming agent, gelatin tannate), a statistically significant reduction in the number of stools was observed at 12 hours posttreatment in patients treated with the combination compared with patients receiving ORS alone [13].

A recent case report of a 4.5-month-old baby girl with a 2-day history of watery diarrhea and fever due to rotavirus gastroenteritis demonstrated that the administration of a film-forming agent in combination with intravenous fluid therapy was able to considerably improve the child's diarrhea within the first 12 hours and diarrhea resolved completely within 3 days [15].

Therefore, it appears that there are currently enough data to support the use of these film-forming agents in combination with ORS to stop diarrhea, particularly in the pediatric population. Moreover, as already reported in adults [10], despite some between-group baseline differences which may reflect selection bias, xyloglucan showed a trend to improve other related symptoms including nausea, vomiting, and abdominal pain, probably due to the favorable effects of xyloglucan on the intestinal mucosa.

The favorable results obtained in adults and in children, in the present study, represent the first demonstration at clinical level of the efficacy and safety of xyloglucan, although further randomized double-blind clinical trials will be performed to confirm these results, using larger samples. We would also note the difficulties in recruiting children for randomized clinical trials performed according to ICH guidelines.

Finally, no adverse events were recorded during our study, thus further supporting the safety profile of this family of agents.

In conclusion, the administration of xyloglucan in combination with ORS is an efficacious and safe option in clinical practice for the treatment of acute diarrhea in the pediatric population, with a rapid onset of action in reducing types 6 and 7 stools. The results obtained in this study support the use of xyloglucan added to ORS in children with acute gastroenteritis.

## Figures and Tables

**Table 1:** Baseline demographic and clinical characteristics of children.

	Statistical variable	Xyloglucan + ORS (*n* = 18)	ORS (*n* = 18)	Total (*n* = 36)
Gender (girls)	*n* (%)	9 (50.00)	12 (66.67)	21 (58.33)
Age (years)	Mean (SD)	4.72 (4.33)	3.94 (3.26)	4.33 (3.80)
Age ranges	*n* (%)			
≤1 year		3 (16.67)	2 (11.11)	5 (13.88)
1–5 years		7 (38.89)	10 (55.56)	17 (47.22)
5–10 years		4 (22.22)	5 (27.78)	9 (25.00)
>10 years		4 (22.22)	1 (5.56)	5 (13.88)
Hydration status	*n* (%)			
Normal		11 (61.11)	13 (72.22)	24 (66.66)
Mild dehydration		7 (38.89)	5 (27.78)	12 (33.33)
Body temperature				
≤37°C		9 (50.00)	7 (38.89)	16 (44.44)
37-38°C		5 (27.78)	6 (33.33)	11 (30.55)
>38°C		4 (22.22)	5 (27.78)	9 (25.00)

ORS: oral rehydration solution; SD: standard deviation.
